# Effects of probiotics on *Zebrafish* model infected with *Aeromonas hydrophila*: spatial distribution, antimicrobial, and histopathological investigation

**DOI:** 10.1186/s12866-022-02491-4

**Published:** 2022-06-27

**Authors:** Sheida Ehsannia, Hamed Ahari, Shapour Kakoolaki, Seyed Amirali Anvar, Shima Yousefi

**Affiliations:** 1 Department of Food Science and Technology, Science and Research Branch, Islamic Azad University, Tehran, Iran; 2 Agricultural Research Education and Extension Organization (AREEO), Iranian Fisheries Science Research Institute, Tehran, Iran; 3 Department of Food Hygiene, Science and Research Branch, Islamic Azad University, Tehran, Iran; 4 Faculty of Agricultural Technology and Engineering, University of Tehran, Tehran, Iran

**Keywords:** Immunomodulation, Intestinal Tissues, Probiotic Strains, Spatial Distribution, *Zebrafish*

## Abstract

Usage of “probiotics” for treatment of food-borne pathogens associated diseases, makes a significant reduction in transmission of resistant bacteria, and antimicrobial resistance genes from aquaculture environments to humans. In this research, the authors aim to evaluate the immunomodulatory, and histological effects of two probiotic strains on the *Zebrafish* model. Fish models were treated with *Lactobacillus delbrueckii* (G2), *Lactobacillus acidophilus* (G3) and both probiotics (G4) and compared with the control group (G1) (only infected by pathogen and receiving no probiotic). Biometric tests, height, weight, and mortality rate of the fishes were assessed. Afterward, RT-PCR was conducted for bacterial existence of probiotic strains, and quantitative assessment of alterations in targeted immune genes. Subsequently, histological sampling was done for investigation of spatial distribution, and villus length in proximal, middle, and distal sections of intestinal tissues. Based on the results, G4 showed the highest gene expression for *Lactobacillus acidophilus* after 28 days (*P* < 0.05). G4 also showed an increase in the number of goblet cells and villus length in the middle and distal sections of intestinal tissue after 56 days. Furthermore, after 56 days, the highest number of intraepithelial cells was observed in the proximal sections of intestinal tissue in G4. G2 and G3 showed significant differences in comparison with G1 (*P* < 0.05). After 60 days, the highest gene expression for *Lactobacillus bulgaricus* was found in group treated with only this probiotic bacteria. The highest expression level of IL-1β and TNF-α were found in G1. The highest survival rate was in the case of groups only treated with *Lactobacillus bulgaricus* (G2). To sum up, it seems that usage of probiotics for the improvement of public health and fisheries industries can be helpful.

**Graphical Abstract:**

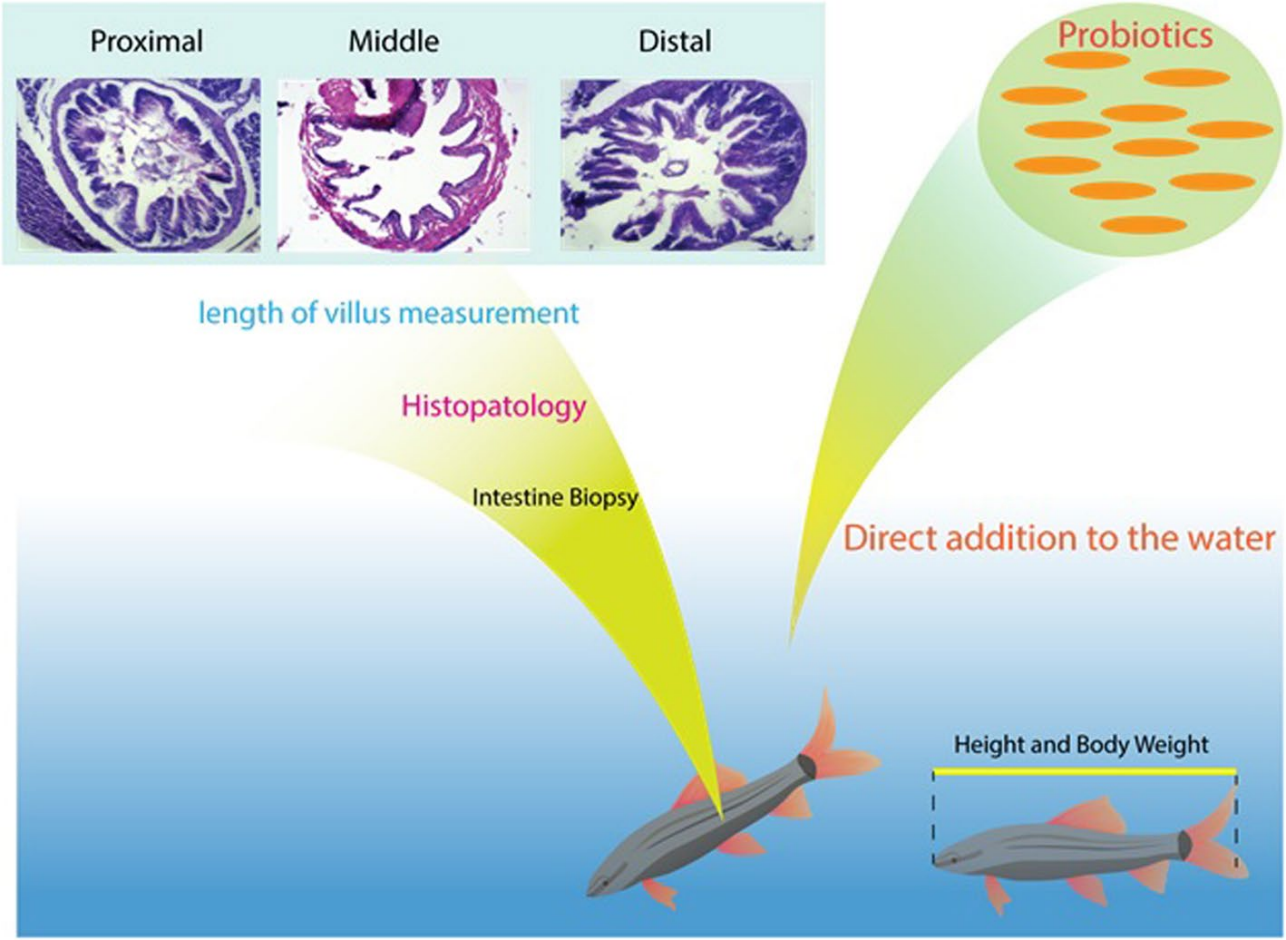

## Introduction

Marine products are known as the part and parcel of the human food basket [1]. It is rational to do a comprehensive look at the need for the treatment of food-borne pathogens-associated diseases, especially the ones related to fisheries industries [2–7]. Controlling infectious diseases in aquatic organisms can majorly contribute to promoting the healthiness of human beings [8], improve public health, and reduce the rate of Gastrointestinal (GI) disorders in marine organisms and humans like colitis or Inflammatory Bowel Diseases (IBD) [6, 7, 9].

The fish are susceptible to many bacterial, viral, fungal, and parasitic pathogens. In the genera of freshwater and brackish water fish, *Infectious Hematopoietic Necrosis Virus* (*IHNV*), *Lymphocystis Disease Virus* (*LCDV*), *Pseudomonas, Citrobacter*, *Proteus, Streptococcus, Edwardsiella, Staphylococcus,* and different species of *Vibrio*, are considered as the most life-threatening infections. One of the major diseases in the fish is caused by *Aeromonas hydrophila*, which is colonized in the GI tracts of the fish, leading to severe hemorrhagic septicemia and enteritis [10]. Current therapeutic strategies for the fish mainly include the usage of chemotherapeutic drugs like antibiotics and vaccination [5]. Alteration in gut microflora, the inadequacy of vaccines on some fishes due to small size and high mortality rate compared with the big ones, the emergence of antibiotic-resistant bacterial strains, ecological contamination in the fish habitats, all impose extortionate expenditure that makes basic medical researchers and food science technologists toward more efficient approaches [2, 4, 11, 12].

One of the most newly introduced approaches goes back to the usage of “Probiotics” as an alternative strategy for making a meaningful decrease in the usage of antibiotics [13], reduction in the re-emergence of antibiotic-resistant strains, a significant decline in the emergence of new human pathogens, as well [14]. As Food and Agriculture Organization (FAO) and World Health Organization (WHO) defined probiotics are living microorganisms with beneficial effects on the health and well-being of the host when orally administered in sufficient amounts [3, 14].

There are a wide array of researches that highly recommend dietary supplementation with digestible food additives like probiotic bacteria which are classified as health-promoting bacteria. These bacteria can attenuate the pathogenicity of GI strains and exacerbate the antibody responses like Immunoglobulin A (IgA), and IgG for preventing infections by affecting intestinal epithelial and Dendritic Cells (DCs). Otherwise, some strains can induce secretion of Interleukin-6 (IL-6) and Tumor Growth Factor-β (TGF-β) from DCs, enhance IL-12 secretion for Natural Killer (NK) cells activation, and/or inhibit the production of Tumor Necrosis Factor‐α (TNF‐α) through a Toll-Like Receptor-2 (TLR-2) dependent performance, leading to suppression of NF‐κB activation. It is worth mentioning that some of the isolated LAB strains with human intestinal tracts resource have depicted an acceptable probiotic efficacy in animals [2, 7, 15–22].

Even though alteration of the microbiome through the application of probiotics for animals is fairly a new concept, it seems that these probiotics can be of high prominence for aquatic organisms, drawing new horizons of microbial biotherapy for marine organisms, as well. In fish farming, many published studies have demonstrated profound effects of probiotics for various fish species, including *rainbow trout* [23], common carp [5, 24], finfish [25], Mediterranean species [26], *Mozambique tilapia*, tilapia *(Oreochromis niloticus*) [27, 28], juvenile hybrid tilapia [29]*, Nile tilapia *[30, 31]*, European Sea bass juveniles*, grouper fish [32], gilthead sea bream (*Sparus aurata*) [33], Caspian white fish (*Rutilus frisii kutum*) [34], striped catfish (*Pangasianodon hypophthalmus*) [35], African catfish (*Clarias gariepinus*) [36], Shabot (*Barbus grypus*, and *Tor grypus*) [37, 38], grass carp [39, 40], black swordtail (*Xiphophorus helleri*) [41], eastern oyster (*Crassostrea Virginica*) [42], *zebrafish* (*Danio rerio*) [43–47], and shrimp [6, 48–51].

There are several well-characterized and potentiated strains that act as probiotics, altering the composition of intestinal microbiota to achieve favorable effects such as enhancing growth, reproduction, digestion, nutrient absorption and metabolism, intestinal mucosal morphology, competitive binding and blocking of adhesion sites, host defense and immunity against infections, and disease resistance of the host organism. Although, their usage and mechanisms of action are yet to be understood and completely legalized [2, 3, 11, 17, 52–54].

There are untackled obstacles including difficulties in bacterial viability after ingestion and amplification of probiotics interaction with GALT cells. Thereafter, advocating such theories are almost impossible to be guaranteed [15–17]. On the other hand, type of administration method (as water or food additives, single or combinative administration, duration of feeding, encapsulated or enriched ones, live or dead/inactivated bacteria, locality to the host or culturing environment) should not be underestimated [4, 53, 55, 56]. Therefore, comprehensive research to fully characterize the fish intestinal microbiota, and their mechanisms of action on the intestinal ecosystem, immunity, fish health, and performance, and optimizing the most efficient dosage as food additives are necessitated to manipulate gut microbiota. Unquestionably, validation of up-to-date and innovative molecular procedures to study the gut microbiota through in vitro, ex vivo*,* and in vivo models can decipher mentioned ambiguities, and qualify commercialization of aquaculture production.

Zebrafish are considered ornamental and aquarium fish due to their good habituation, they are easy to maintain, and their natural habitat has been reported to be freshwater from the tropics. The body shape of the zebrafish is slender and long and has golden and blue stripes that are stretched along the body and tail of the animal and the male and female sexes of this fish are separated and can be easily identified. Unlike mammals, zebrafish do not have tissue beneath the mucous membrane. In zebrafish, parts of the intestine are differentiated based on morphology and gene expression: the anterior part of the intestine (Proximal), the middle part, and the posterior part of the intestine (Distal). Due to this separation, digestive enzymes are strongly expressed in the anterior part of the intestine.

Totally, in this experimental study, authors aimed to investigate the immunomodulatory properties and spatial distribution of probiotic bacteria (*Lactobacillus acidophilus (LA)* and *Lactobacillus delbrueckii (LD)*) on GI tissues of a *Zebrafish* model infected by *Aeromonas hydrophila* through histopathological sampling and quantitative assessment of targeted immune genes involved in immunomodulation (IL-1β and TNF-α).

## Material and methods

### Ethical considerations

This experimental study accessed ethics approval from the Science and Research Branch, Islamic Azad University Research Ethic's Committee (no.)

### Study design and procedure

#### Preparation of *Zebrafish*

To prepare the most suitable aquarium for our studied population of fish, 12 aquariums and 12 air pump were bought. After washing, the aquarium container was watered and decolorized. Ventilation pumps were installed for all of them, as well. Constant water temperature with continuous aeration was considered. Water was exchanged daily before feeding. 240 Zebrafish were bought from a fish farm and kept inside the aquariums (Temperature: 26 ± 2, pH: 7–7.5, light: 12:12, and salinity: 0.25–0.75 ppt). Feeding was done by commercialized food commencing from one week before the main test (during the acclimatization period). Probiotic-based food was considered in our study during our assay. All fish experiments were carried out based on the Regulations for Animal Experimentation at Jilin Agricultural University (JLAU08201409).

#### Preparation of probiotic bacterial strains

*LA* and *LD* strains subspecies of *Lactobacillus bulgaricus* were dedicated by the microorganism bank of Iranian Biological Resource Center (ATCC® 4356™, and ATCC® BAA2844™, respectively). Isolation and suspension of both mentioned probiotic strains were made from the culture of both strains. Then, a loop of both bacteria was inoculated into MRS broth (Merck Co, Germany), and anaerobically incubated at 37ºC in an atmosphere of 5% CO_2_ for 24 h on a shaking incubator with 180 rpm. After achieving enough turbidity and optimized concentration of bacterial growth, 100 ml of bacterial suspension was centrifuged for 15 min at 4000 rpm and the supernatant was removed. Then, the bacterial pellet was dissolved in normal saline (pH = 7.5). Thereafter, 0.5. McFarland Standard was prepared to adjust the turbidity of bacterial suspension (0.05 mL of 1.175% barium chloride dihydrate with 9.95 mL of 1% sulfuric acid). Here, viable counts of cultured bacteria as Colony Forming Unit (CFU) measured at Optical Density _600 nm_ (OD_600nm_) was equivalent to 1 × 10^8^. They were aliquoted in 15 cc falcons, and after adding glycerol 15% v/v (Merck Co, Germany), stored temporarily at the freezer with -20ºC until further usage in the next steps [57].

#### Preparation of Pathogenic Bacterial Strain

*Aeromonas hydrophila* strain was dedicated by *the* microorganism bank of the Iranian Biological Resource Center (ATCC® 7966™). After transferring this bacterial culture Petri dishes into our well-equipped microbiology laboratory, isolation and suspension of mentioned strains were made from a culture of strain. Then, a loop of bacteria was inoculated into Luria–Bertani (LB) Broth (as an enrichment medium) (Merck Co, Germany), and a serial dilution was made. Then, confirmed bacteria were cultured in Nutrient (NA) Agar (Merck Co, Germany). Thereafter, 0.5. McFarland Standard was prepared as mentioned earlier to adjust the turbidity of the bacterial suspension. Here, viable counts of cultured bacteria (in the stationary phase) as CFU measured at OD_600nm_ were equivalent to 1.3 × 10^8^ CFU g^−1^. Bacteria were resuspended and transferred to tanks at a final concentration of 1.3 × 10^8^ CFU g^−1^. Of note, making an infectious model for the studied population of *Zebrafish* was measured out 56 days after starting our study [58].

#### Feeding

Feeding was done through commercialized food of KOI & GOLDFISH (pellet) twice a day (11.00 AM and 5.00 PM) for the control group. For case groups, feeding was done through probiotic-based food twice a day (11.00 AM and 5.00 PM). The amount of food was estimated as 2% of the overall weight of the fish in both groups. The mean weight of the fish was assessed through M.T.ELECTRONIC BALANCE. The amount of needed food was calculated according to the number of fish in each aquarium and the mean weight of the fish in every round of feeding.

According to the weight of needed food, probiotics were added as 1%-2% of food which was calculated 0.0003 g. (As one milliliter of bacteria is equivalent to 500 mg (mg) of the dried pellet of bacteria). To preserve the powdered phase of each pellet, skimmed milk was mixed with them. The formulation for preparation of each food is as follows:

$$Three gram skimmed milk + 100-gram food pellet+1-2 gram probiotics$$

It is worthy to mention that one of the variables in this study is the detection of the spatial distribution of probiotics in the intestinal tissues of the fish. So, the fluorescent dye was added to the probiotic-based food formula to be traced.

#### Treatments and groups

In this study, a random distribution was done for the fish. In each aquarium, 20 *Zebrafish* were considered. Hence, the studied population of the fish was divided into four groups as follows with three repetitions:

- **G1:** Control group: *Zebrafish*infected with*Aeromonas hydrophila*and receiving no probiotic bacteria.
- **G2:** Zebrafish infected with Aeromonas hydrophila and receiving only 1×10^8^ CFU of Lactobacillus delbrueckii.
- **G3:** *Zebrafish* infected with *Aeromonas hydrophila* and receiving only 1×10^8^ CFU of *LA*.
- **G4:** *Zebrafish* infected with *Aeromonas hydrophila* and receiving 1×10^8^ CFU g^-1^ of both probiotic bacteria.

#### Biometric tests

From the 0^th^ day of the test, the height and weight of the *Zebrafish* were measured by a ruler and M.T.ELECTRONIC BALANCE, respectively. The mortality rate of each treated group was registered since 0^th^ day of starting our study (feeding). In this regard, the number of dead fish was recorded at the end of each week.

#### Histopathological study (Hematoxylin & Eosin (H&E))

Three fish were collected randomly from each treated group. Under the biological hood and aseptic condition, the anatomical site of the intestine was dissected and intestinal tissues were separated. Samples were fixed with formalin 10% for 24 h. In the next step, samples were put in ethanol for dehydration with ascending degrees as 50 min in 70º, 50 min in 80º, 50 min in 90º, and 50 min in 100º, respectively. Next, xylol type I (45º) and II (pure) was used for substitution with ethanol for 40 min. Then, to remove xylol, samples were soaked and infiltrated in melted paraffin I and II (70ºC) for 40 min. After paraffin freezing, samples were placed in embedded melted paraffin to be prepared for sectioning by microtome (5–10 micron). Afterward, to melt paraffin, prepared lams were put in oven 90ºC for 20 min. To have a transparent sample and remove paraffin from the samples, xylol type I (45º) and II (pure) was used for 10 min. Now, samples were put in ethanol, for hydration and removing ethanol with descending degrees as one minute in 100, one minute in 90, one minute in 80, one minute in 70, and one minute in tap water. In the end, samples were dyed with Hematoxylin as basophil dye for 15 min and then were washed. In the end, they were put in Eosin for 10 s. In this stage, dehydration by ascending degrees of ethanol, and infiltration by xylol were done as mentioned earlier. After doing a montage with Entellan for sticking lam and lamels and removing air bubbles, lams were prepared for being investigated by fluorescent microscopes [59, 60].

#### Detection of probiotics in the intestinal tissues

By a random selection, three fish were collected from each treated group. Under the biological hood and aseptic condition, the anatomical site of the intestine was dissected and intestinal tissues were separated. Samples were kept in formalin 10%. As it was mentioned earlier, fluorescent dying was used for the detection of probiotics in the intestinal tissues and investigation of spatial distribution. Rhodamine (green for *LA*) and Dil (red for *Lactobacillus delbrueckii*) dyes were formulated in the preparation of each probiotic-based food (wavelength = 520 nm). The formulation for preparation of each dye in the food pellet is as follows:

$$1\upmu of each dye+ 1\upmu of suspended bacteria+ 1000\upmu L of Phosphate Buffer Saline (PBS)$$

After incubation of these solutions for five minutes at room temperature, dark vials containing mentioned solution were incubated for 20 min at the refrigerator temperature. Fluorescence microscopy-based approaches were used for the detection of formulated probiotic bacteria.

#### Detection of probiotics through PCR

To quantitatively assess the expression levels of each probiotic in the intestinal tissues, sampling was done on the 28^th^, 56^th^, and 60^th^ day of our assay [61]. Table 1 reveals used primers (forward and reverse primers) according to mentioned sequences for detection of probiotic bacteria through PCR assay.

**Table 1:** Forward and reverse primers used for detection of both probiotic bacteria through PCR

Primer name	Sequence	Length (Amplicon)
Forward primer *L. acidophilus*	GAAAGAGCCCAAACCAAGTGATT	85 bp
Reverse primer *L. acidophilus*	CTTCCCAGATAATTCAACTATCGCTTA	
Forward primer *L. delbrueckii*	CACTTGTACGTTGAAAACTGAATATCTTAA	94 bp
Reverse primer *L. delbrueckii*	CGAACTCTCTCGGTCGCTTT	

#### For quantitative assessment of IL-1β and TNFα genes through RNA extraction and real-time PCR (RT-PCR)

By a random selection, on the 28^th^, 56^th^, and 60^th^ day of our assay, three *Zebrafish* were collected from each treated group. Under the biological hood and aseptic condition, the anatomical site of the intestine was dissected and intestinal tissues were separated. Samples were fixed in formalin 10%, transferred into the molecular laboratory, and kept inside RNase-free vials in the freezer -80ºC until further use in the next steps (RNA extraction).

##### RNA Extraction for Quantitative Assessment of IL-1β and TNFα genes

At the time of assay, samples were removed from the freezer and 300 µl triazole was added to homogenated tissues (next to the ice box). Then RNase-free micro tubes were vortexed for 10 s and kept at room temperature for five minutes. Then, under the biological hood situation, 200 µl of cold chloroform was added and the content of micro tubes mixed gently for 15 s. Next, after keeping micro tubes in 4ºC for five minutes, they were centrifuged for 17 min and 8000 rpm in 4ºC. After watching a clear supernatant, cold isopropanol (volume equivalent to the supernatant) was added to the content of micro tubes and mixed gently. Then micro tubes were incubated at 4ºC for 10 min, which is followed by centrifugation for 12 min and 8000 rpm at 4ºC and resulted in protein denaturation. After removing supernatants, 200 µl of ethanol 80% v/v was added to the pellet to precipitate RNA and micro tubes were vortexed for 10 s. In the next step, centrifugation was conducted for five minutes and 7500 rpm at 4ºC, and supernatants were removed. In this step, a 15-min pause was needed to reach semi-wet pellets. After reaching semi-wet pellets, 30 µl of Di Ethyl Pyro Carbonate (DEPC) water (free of RNase) was added to dissolve RNA precipitation and micro tubes were placed in a water bath in 55ºC-60ºC for 15 min aimed at the complete dissolving of RNA precipitation. Enzymatic properties of RNase-free DNase I were used for removing any DNA contamination. One microliter of MgCl_2_ 10X buffer and 0.5 µl of RNase-free DNase I were added to one microliter of RNA solution and the volume was optimized with DEPC water to 10 µl. After incubating at 37ºC for 30 min, one microliter of EDTA was added to neutralize RNase-free DNase I, and samples were incubated at 65°C for 10 min. Quantity, purity, and integrity of extracted RNA were tested using a spectrophotometer and by electrophoresis on 1% agarose gels as follows [61]. 

##### cDNA synthesis for quantitative assessment of IL-1β and TNFα genes

For quantitative investigation on the concentration of total extracted RNA, the nanodrop-based spectrophotometry approach was measured out and the concentration of RNA was calculated (as ng/µl) in the wavelengths of 260 and 280 nm (260/280). For cDNA synthesis, after confirming about sufficient concentration of RNA, 10 µl of cDNA synthesis reagent was added to 10 µl of treated RNA according to the manufacturer's instructions (Easy cDNA Synthesis kit, Pars tous Co). Then samples were incubated at room temperature (25ºC) for five minutes and subsequently at 60 ˚C for 60 min through thermocycler. Afterward, they were stored on ice boxes and frozen at -20ºC until further steps [61].

##### Designation of Forward and Reverse Primers for Quantitative Assessment of IL-1β and TNFα genes

At the time of assay, Real-Time PCR was recruited for the quantitative assessment of immune genes. Firstly, the genome of Zebrafish was investigated and the GAPDH gene was considered as a standardized (reference) gene. Reverse and forward primers for immune genes (IL-1β and TNFα) were designated by the Gen Runner application (version 6.5) and made by SINACOLON Company. The list of synthesized primers and amplified sequences (Table 2) are as follows:

**Table 2:** Forward and reverse primers used for quantitative detection of reference, and immune genes

Primer name	Sequence	Length (Amplicon)
Forward primer d-GAPDH	CAGAACATCATCCCAGCCTCC	152 bp
Reverse primer d-GAPDH	TTGGCAGGTTTCTCAAGACGG	
Forward primer d-IL-1β	ACAGCACACACACTGATGCAC	218 bp
Reverse primer d-IL-1β	AGAATAAGCAGCACTTGGGGA	
Forward primer d-TNF-α	TGGATTGTGAACGAAAGTGAG	108 bp
Reverse primer d-TNF-α	AGCAATGTTCAGATGTGTTGG	

To refrain from any homologies and complementary sequences with nucleotide sequences of other genes, mentioned primers were rechecked in BLAST search tool of National Center for Biotechnology Information (NCBI) website in (http://www.ncbi.nlm.nih.gov/Blast). For estimation of our target immune genes/reference gene ratio, efficiency for PCR is hypothesized 100%. So, this formula was considered:

$$\mathrm{Ratio}=2^{-\left(\triangle\mathrm{CTcase}\;-\;\triangle\mathrm{CTcontrol}\right)}$$

##### RT-PCR for quantitative Assessment of IL-1β and TNFα genes

The name of the reagents and their used volumes for each reagent was mentioned in Table 3:

**Table 3:** Reagents and used volume for quantitative assessment of IL-1β and TNFα genes

Reagents	Volume (Total Volume 10 µl)
Real Q Plus 2 × Master Mix Green (HIGH ROX) Including: KCl, MgCl2, dNTP, taq DNA polymerase	5 µl
cDNA	2 µl
Forward Primers	0.5 µl
Reverse Primers	0.5 µl
DEPC water	2 µl

Stages of RT-PCR (cycles and temperature) was mentioned below in Table 4.

**Table 4:** Cycles and temperature in RT-PCR assay for quantitative assessment of immune genes

Step	Temperature	Time	Cycles
Initiation	95	15 min	One
Denaturation	95	15 s	40
Ligation	60	30 s	40
Elongation	60	30 s	40

### Bio-statistical analysis of data

Prism software and ANOVA statistical tests were used to analyze the obtained results. All tests were performed in triplicates and results were expressed as Mean ± Standard Deviation (Mean ± SD). Overall statistical significant difference level was considered as p value < 0.05.

## Results

### Histopathological analysis

**Fig. 1:**
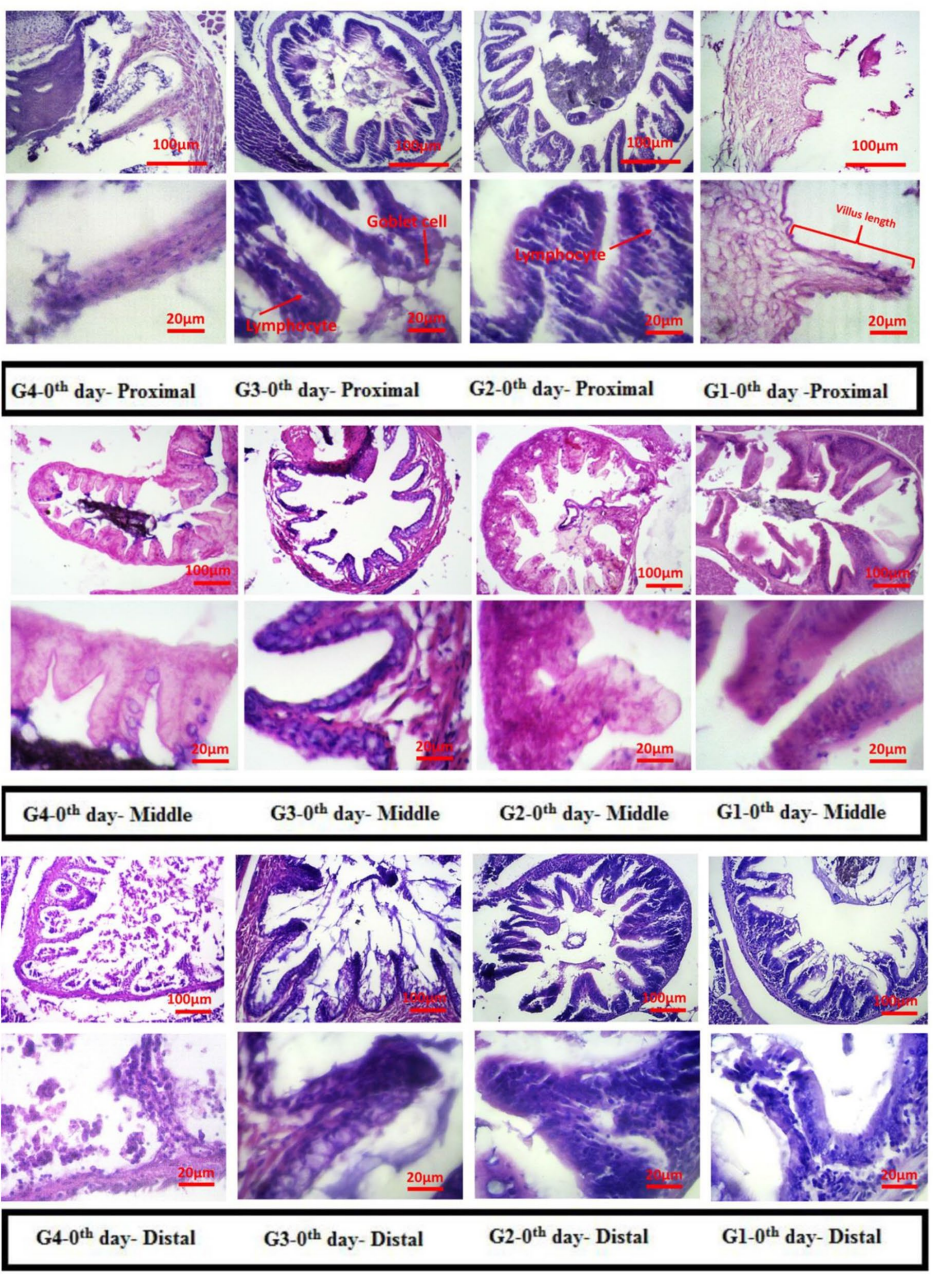
Histopathological changes in intestinal tissues of *Zebrafish* in 0^th^ day of assay

**Fig. 2:**
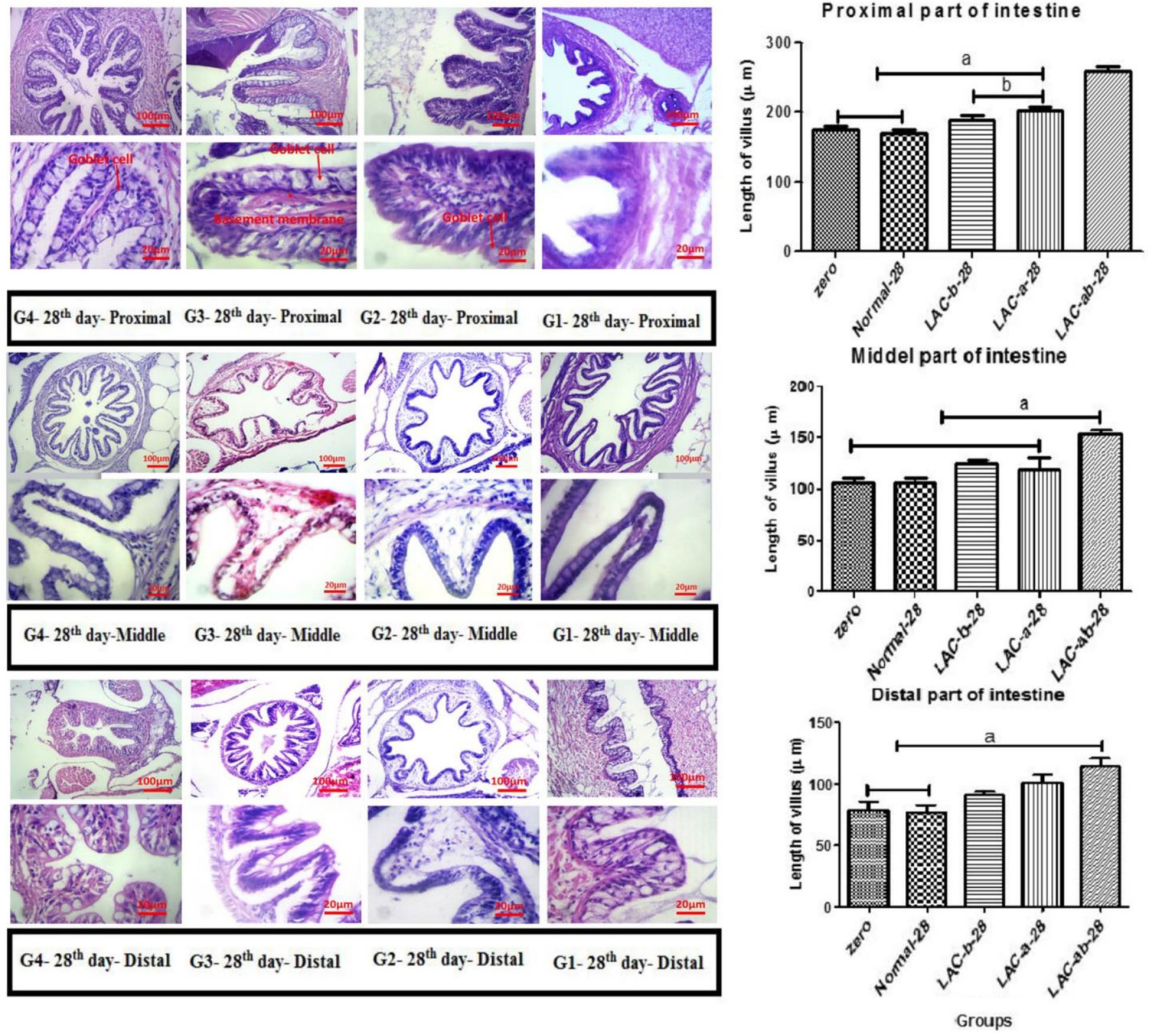
Histopathological changes and villus length in intestinal tissues of *Zebrafish* on the 28^th^ day. A) Proximal, B) Middle, and C) Distal part of the intestine

**Fig. 3:**
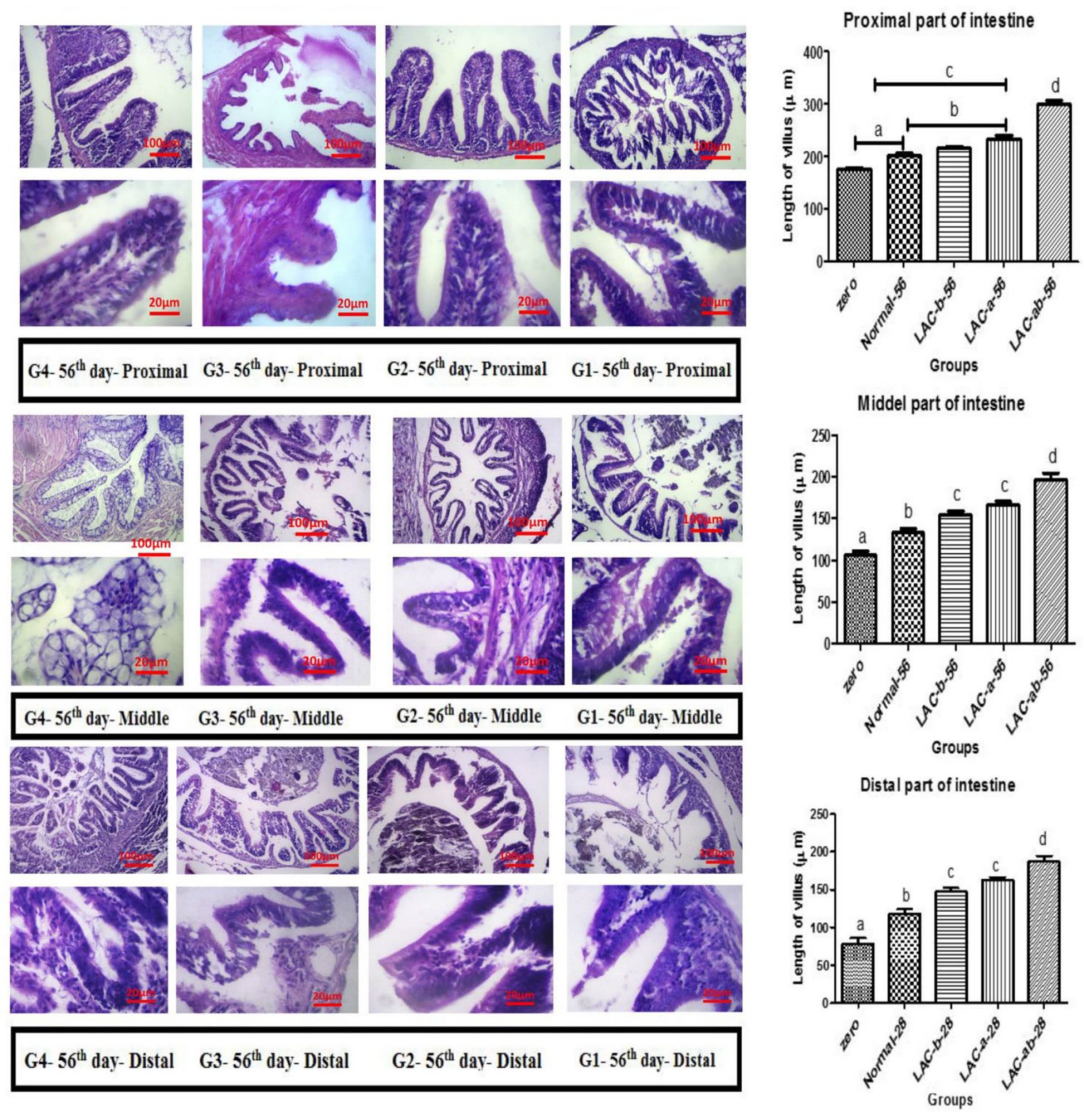
Histopathological changes and villus length in intestinal tissues of *Zebrafish* on 56^th^ day. A) Proximal, B) Middle, and C) Distal part of the intestine

**Fig. 4:**
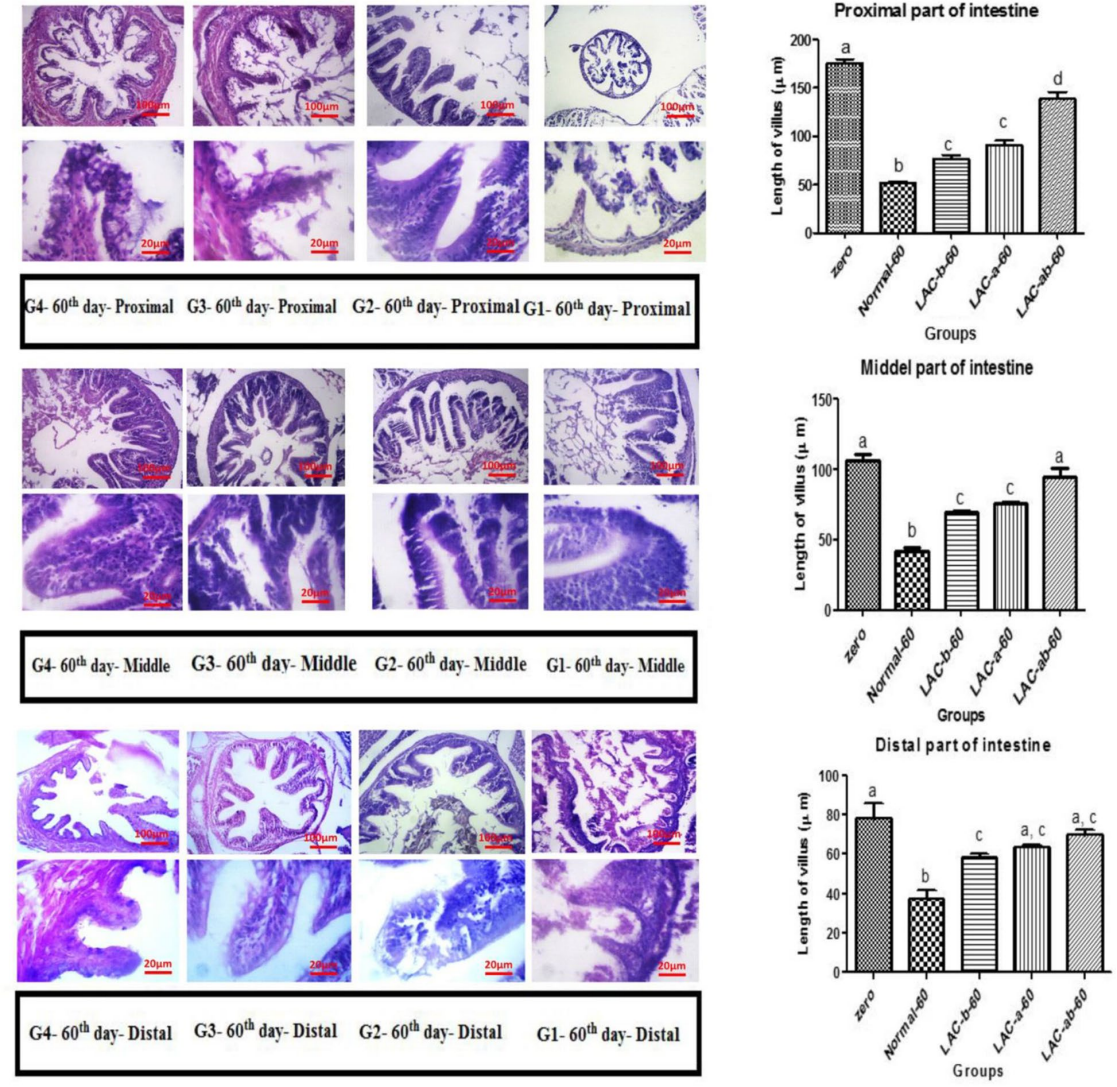
Histopathological changes and villus length in intestinal tissues of *Zebrafish* in 60^th^ day. A) Proximal, B) Middle, and C) Distal part of the intestine

Figures (Fig. 1, 2, 3, 4) show results related to histopathological sampling of proximal, middle, and distal sections in intestinal tissues of *Zebrafish* models infected by *Aeromonas hydrophila* in all case and control groups (G1, G2, G3, and G4) in 0^th^, 28^th^, 56^th^, and 60^th^ day of assay.

Figure 1, depicts histopathological changes in proximal, middle, and distal sections in intestinal tissues of *Zebrafish* models infected by *Aeromonas hydrophila* in all case and control groups (G1, G2, G3, and G4) on the 0^th^ day of assay.

According to Fig. 1, histopathological sampling of proximal, middle, and distal sections of intestinal tissues through H&E dying on the 0^th^ day of the assay for all case and control groups, did not show any pathological signs.

Considering the proximal part of the intestine (Fig. 2A) it can be seen that no significant difference was observed in the normal group in comparison with its initial form (G1). Considering the treated groups, G4 showed longer villus compared to G2 and G3 (*P* < 0.01). Comparing cases treated with just one probiotic, G3 showed a significant difference compared to the G2 (*P* < 0.05). Considering the villus length in the middle section (Fig. 2B), the zero group and G1 showed no significant difference (*P* > 0.05). G2 and G3, with no significant difference towards each other (*P* > 0.05), depicted a difference compared to the G1 (*P* < 0.05). G4 showed a significant difference in comparison to the other groups (*P* < 0.05). Regarding the changes in the villus length in the distal section, G1 showed no significant difference in comparison with the zero group. After treatement with probiotics, the length of villus increased significantly in G2 (*P* < 0.05). interestingly, G3 showed longest villus than G2 (*P* < 0.05). G4, in which fishes were treated with both probiotics, depicted a significant difference in comparison with other groups (*P* < 0.05).

On the one hand, to make a comparison between the G2, G3, and G4, there was an insignificant increased rate of intra-epithelial lymphocytes in the G2 than G3 and G4. On the other hand, there was a significant difference between the G2 and G3 and G4 in proximal, and middle sections in intestinal tissues of *Zebrafish* models. The proximal section had the longest villus, while the small one belonged to the distal section.

The number of goblet cells was increased in all parts of the intestine compared to the control group. This increase in the number of cells was observed in the group of fish treated with both probiotics (G4) significantly higher than the other groups (G2 and G3). A significant number of goblet cells were also observed in the middle part of the intestine relative to the proximal and distal.

According to Fig. 3, there was an increased rate of goblet cells in proximal, middle, distal sections in intestinal tissues of *Zebrafish* models with a significant difference among G3 & G4 and control groups (G1) after 56 days (P < 0.05).

Considering the proximal section, there was an increased length of the intestinal villus in G2, G3, and G4 after 56 days of the assay in comparison with zero and control groups (G1). After 56 days, the normal group showed a significant increase in the villus length compared to the zero group (*P* < 0.05). No significant difference was observed between G2 and G3 (*P* > 0.05), but both showed a significant increase in the villus length compared to the G1. G4 also showed a significant difference in comparison with all groups (*P* < 0.01). A similar trend was observed for the middle and distal sections. Villus in the proximal section had the longest length compared to that of the middle and distal sections.

There was also a significant difference in intraepithelial lymphocytes of the proximal, middle, and distal sections of *Zebrafish* models in G4 in comparison with the control group. The number of goblet cells was increased in all parts of the intestine compared to the control group.

According to Fig. 4, the normal group showed a significant increase in the villus length in all sections compared to the zero group (*P* < 0.05). G2 showed a significant difference in comparison with the G1, while showed no significance compared to G3. it has been demonstrated that there was a decreased rate of intestinal villus with a significant difference between control groups (G1) and probiotic receiving groups (G2, G3, and G4) after 60 days of the assay. Also, there was a decreased rate of an intestinal villus in Zebrafish models infected by *Aeromonas hydrophila* treated with probiotic bacteria (G3, G4) with a significant difference compared to zero groups after 60 days of the assay. Interestingly, there was an increased rate of intra-epithelial lymphocytes in Zebrafish models infected by *Aeromonas hydrophila* treated with both probiotic bacteria (G2) with a significant difference in comparison with other groups after 60 days of the assay. There was a decreased rate of goblet cells in proximal, middle, and distal sections of intestinal tissues of Zebrafish models infected by *Aeromonas hydrophila* treated with probiotic bacteria (G3, G4) in comparison with control groups (G1) three days after induction of infectious by *Aeromonas hydrophila*.

Totally, according to the acquired results, from assessment on 0th, 28th, 56th, and 60th day of treatment with probiotics in all control and case groups, it can be summarized that there was an increased rate of intestinal villus length in middle, and distal sections of intestinal tissues of Zebrafish models infected by Aeromonas hydrophila treated with both probiotic bacteria (G2) with a significant difference in comparison with other groups after 28 and 56 days of the assay. It is worth mentioning that there was an increased rate of intra-epithelial lymphocytes in proximal sections of intestinal tissues of Zebrafish models infected by Aeromonas hydrophila treated with both probiotic bacteria (G2) with a significant difference in comparison with other groups after 56 days of the assay.

### Investigation of spatial distribution

**Fig. 5:**
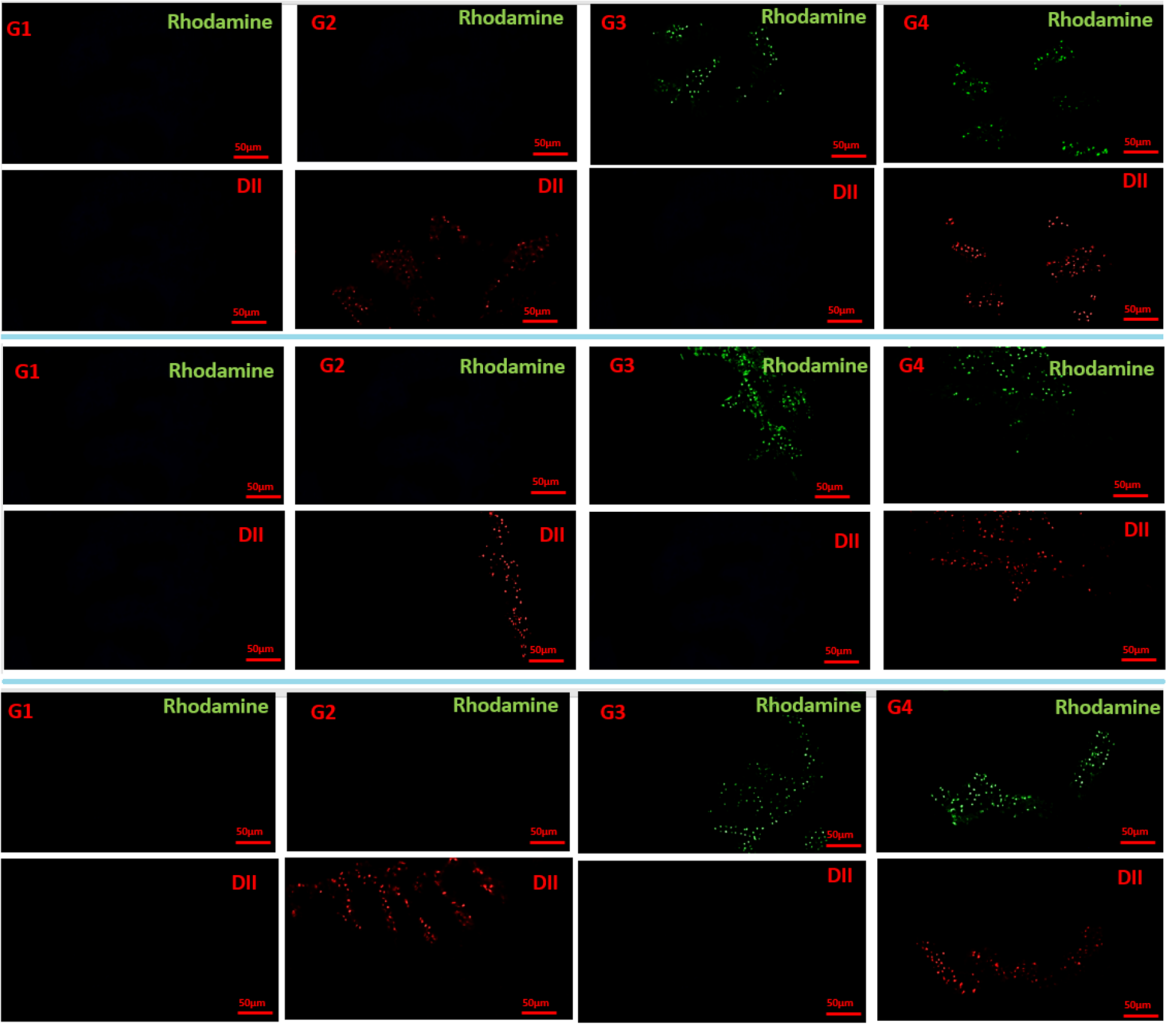
Dil staining for the investigation of the spatial distribution of all groups after A) 28 days, B) 56 days, and C) 60 days

Figure 5, shows results related to the investigation of spatial distribution after fluorescent dying (Rhodamine and Dil) for all case and control groups (G1, G2, G3, G4).

### Quantitative assessment of both probiotics in the intestinal tissues through real-time PCR in 28^th^, 56^th^, and 60^th^ day of Assay

**Fig. 6:**
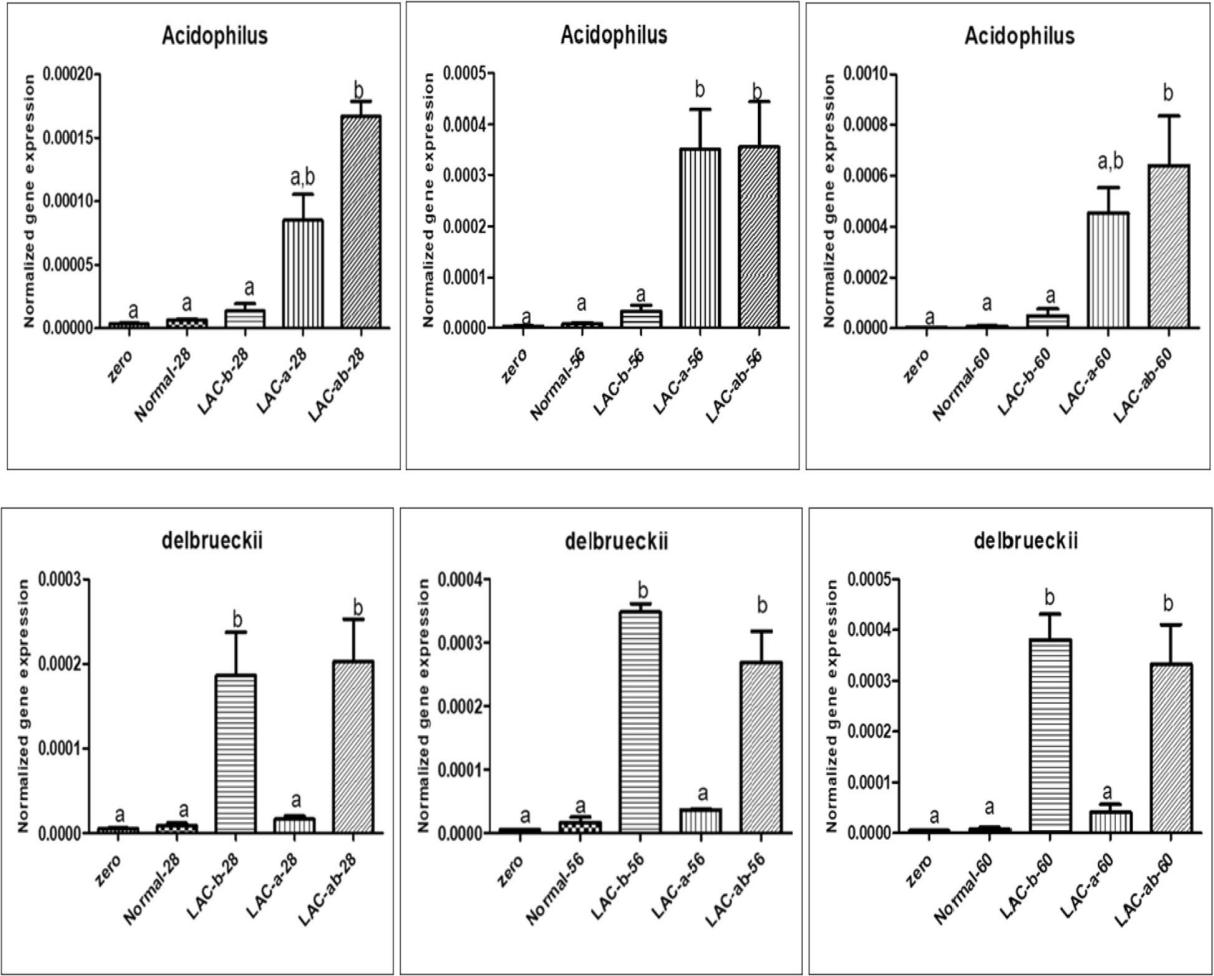
Quantitative investigation of probiotics in the A) 28^th^, B) 56^th^, and C) 60^th^ day of assay

Figure 6, depicts results related to quantitative assessment of both probiotics in intestinal tissues of *Zebrafish* models infected by *Aeromonas hydrophila* in all case and control groups (G1, G2, G3, and G4) on the 28^th^, 56^th^, and 60^th^ day of assay. Here, all of the results were reported in comparison with control groups (G1).

On the one side, according to Fig. 6, results of a quantitative assessment of *LA* in the intestinal tissues through Real-Time PCR on the 28^th^ day of the assay, show that the existence of mentioned probiotic was not found (or very low existence) in zero group. On the other side, the most abundant existence of *LA* was found in intestinal tissues of *Zebrafish* models infected by *Aeromonas hydrophila* receiving both prebiotic bacteria (G4) in comparison with control and zero groups. There were not any significant differences among zero, control (G1), and cases groups receiving only one type of probiotic bacteria (G3, G2).

Results of a quantitative assessment of *LD* in the intestinal tissues through Real-Time PCR on the 28^th^ day of assay, show that the existence of mentioned probiotic was not found (or very low existence) in zero, control, and cases receiving only one type of probiotic bacteria group (G2) without any significant difference. The most abundant existence of *LD* was found in intestinal tissues of *Zebrafish* models infected by *Aeromonas hydrophila* receiving both prebiotic bacteria (G4), and cases receiving only one type of probiotic bacteria group (G3) in comparison with control, zero, and cases receiving only one type of probiotic bacteria group (G2) groups. There was an increased rate for the existence of *LD* in intestinal tissues of *Zebrafish* models infected by *Aeromonas hydrophila* receiving both prebiotic bacteria (G4) compared to cases receiving only one type of probiotic bacteria group (G3) without any significant difference.

Results of a quantitative assessment of *LA* in the intestinal tissues through Real-Time PCR on the 56^th^ day of assay, show that the existence of mentioned probiotic bacteria was not found (or very low existence) in zero, control (G1), and cases receiving only one type of probiotic bacteria group (G2) without any significant difference. Among aforesaid groups, cases receiving only one type of probiotic bacteria group (G3) depict an increased rate for mentioned probiotic bacteria compared to zero, and control (G1) groups without any significant difference. On the other side, the most abundant existence of *LA* was found in intestinal tissues of Zebrafish models infected by *Aeromonas hydrophila* receiving both prebiotic bacteria (G4), and cases receiving only one type of probiotic bacteria group (G2) with a significant difference in comparison with three other groups. Between G4 and G2, there was not any significant difference for the increased rate of *LA.*

Results of the quantitative assessment of *LD* in the intestinal tissues through Real-Time PCR on the 56^th^ day of assay, show that the existence of mentioned probiotic was not found (or very low existence) in zero, control, and cases receiving only one type of probiotic bacteria group (G2) without any significant difference. Among aforesaid groups, cases receiving only one type of probiotic bacteria group (G2) depict an increased rate for mentioned probiotic bacteria compared to zero, and control (G1) groups without any significant difference. The most abundant existence of *LD* was found in intestinal tissues of *Zebrafish* models infected by *Aeromonas hydrophila* receiving both prebiotic bacteria (G4), and cases receiving only one type of probiotic bacteria group (G3) in comparison with control, zero, and cases receiving only one type of probiotic bacteria group (G2) groups with a significant difference. Between these two groups, there was an increased rate for the existence of *LD* in cases receiving only one type of probiotic bacteria group (G3) in comparison with cases receiving both prebiotic bacteria (G4) groups without any significant difference.

Results of a quantitative assessment of *LA* in the intestinal tissues through Real-Time PCR on the 60^th^ day of assay, show that the existence of mentioned probiotic bacteria was not found (or very low existence) in zero, control (G1), and cases receiving only one type of probiotic bacteria group (G3) without any significant difference. On the other side, the most abundant existence of *LA* was found in intestinal tissues of *Zebrafish* models infected by *Aeromonas hydrophila* receiving both prebiotic bacteria (G4), and cases receiving only one type of probiotic bacteria group (G2) without any significant difference. There was an increased rate for the existence of *LA* in cases receiving both prebiotic bacteria (G4), in comparison with zero, control, and cases receiving only one type of probiotic bacteria (G3) group with a significant difference.

Results of the quantitative assessment of *LD* in the intestinal tissues through RT-PCR on the 60^th^ day of assay, show that the existence of mentioned probiotic was not found (or very low existence) in zero, control, and cases receiving only one type of probiotic bacteria group (G2) without any significant difference. Among aforesaid groups, cases receiving only one type of probiotic bacteria group (G2) depict an increased rate for mentioned probiotic bacteria compared to zero, and control (G1) groups without any significant difference. The most abundant existence of *LD* was found in intestinal tissues of *Zebrafish* models infected by *Aeromonas hydrophila* receiving both prebiotic bacteria (G4), and cases receiving only one type of probiotic bacteria group (G3) in comparison with control, zero, and cases receiving only one type of probiotic bacteria group (G2) groups with a significant difference. Between these two groups (G4, and G3), there was an increased rate for the existence of *LD* in cases receiving only one type of probiotic bacteria group (G3) in comparison with all four groups (zero, control, cases receiving both prebiotic bacteria (G4) group without any significant difference.

### Quantitative assessment of IL-1β and TNF-α genes through RT-PCR in 28^th^, 56^th^, and 60^th^ day of assay

**Fig. 7:**
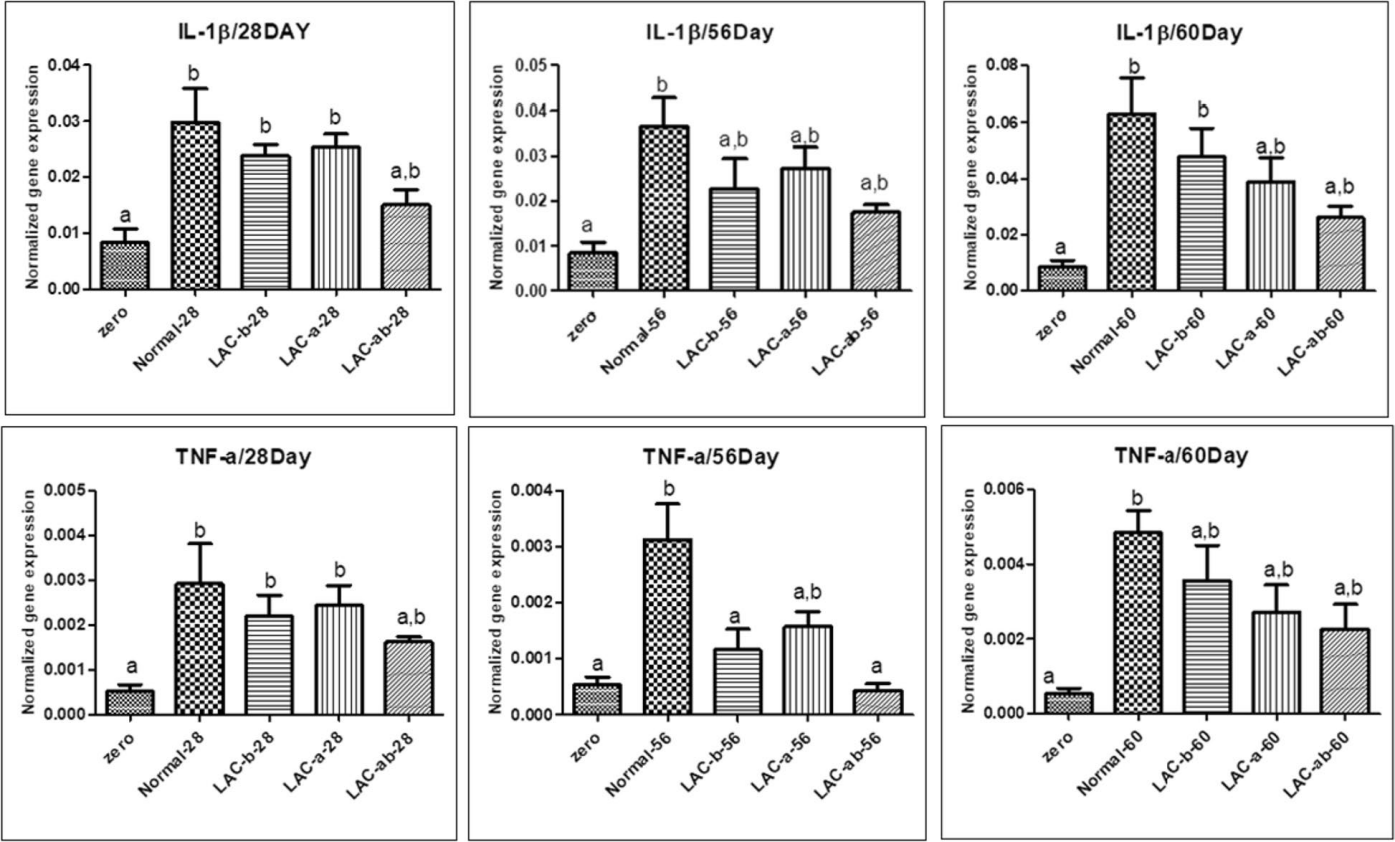
Quantitative investigation of immune genes in the A) 28^th^, B) 56^th^, and C) 60^th^ day of assay

Figure 7, shows results related to quantitative assessment of IL-1β and TNF-α genes as immune target genes in intestinal tissues of *Zebrafish* models infected by *Aeromonas hydrophila* in all case and control groups (G1, G2, G3, and G4) in 28^th^, 56^th^, and 60^th^ day of assay. Here, results are considered as effects of probiotic bacteria on alterations in the expression levels of immune genes according to GAPDH as a reference gene and in comparison with control groups.

According to Fig. 7, lower levels of IL-1β expression are found in zero, and cases receiving both probiotic bacteria groups in comparison with control, and cases receiving only one type of probiotic bacteria (G2, and G3) groups on the 28^th^ day of assay. The lowest and the highest levels of IL-1β expression are related to zero, and control groups, respectively. There was not found any significant difference for levels of IL-1β expression among control, cases receiving only one type of probiotic bacteria (G3, and G2) groups, and cases receiving both prebiotic bacteria (G4) groups, on the 28^th^ day of assay. In addition, there was found an increased level of IL-1β expression with a significant difference in control (G1), cases receiving only one type of probiotic bacteria (G3, and G2) groups compared to zero groups ion the 28^th^ day of assay.

Data related to the 56^th^ day of the assay revealed that the lowest and the highest levels of IL-1β expression are related to zero, and control groups, respectively. There was an increased level of IL-1β expression in cases receiving only one type of probiotic bacteria (G2) groups compared to cases receiving only one type of probiotic bacteria (G3) groups, and cases receiving both prebiotic bacteria (G4) groups without any significant difference. Also, there was not any significant difference in the level of IL-1β expression among control, cases receiving only one type of probiotic bacteria (G3, and G2) groups, and cases receiving both prebiotic bacteria (G4) groups, on the 56^th^ day of assay. Only a significant difference in the level of IL-1β expression was found between the zero, and control groups (*p*-value ≤ 0.05).

Investigation of data acquired from the 60^th^ day of the assay (three days after making our *Zebrafish* models infected by *Aeromonas hydrophila*) indicated that the lowest and the highest levels of IL-1β expression are related to zero, and control groups, respectively. Among cases receiving only one type of probiotic bacteria (G3, and G2) groups, and cases receiving both prebiotic bacteria (G4) groups, the highest levels of IL-1β expression was related to cases receiving only one type of probiotic bacteria (G3) groups and no significant difference was found. Similarly, there was not found any significant difference for levels of IL-1β expression among control, cases receiving only one type of probiotic bacteria (G3, and G2) groups, and cases receiving both prebiotic bacteria (G4) groups on the 60^th^ day of assay. Only, there was found a significant difference for levels of IL-1β expression between control, and cases receiving only one type of probiotic bacteria (G3) groups, with zero group on the 60^th^ day of assay.

Investigation of data acquired from quantitative assessment of TNF-α on the 28^th^ day of assay revealed that the lowest and the highest levels of TNF-α expression are related to zero, and control groups, respectively. Among cases receiving only one type of probiotic bacteria (G3, and G2) groups, and cases receiving both prebiotic bacteria (G4) groups, the highest levels of TNF-α expression was related to cases receiving only one type of probiotic bacteria (G2) groups and no significant difference was found. Similarly, there was not found any significant difference for levels of TNF-α expression among zero, and control groups, with cases receiving only one type of probiotic bacteria (G3, and G2) groups, and cases receiving both prebiotic bacteria (G42) groups on the 28^th^ day of assay. There was not found any significant difference in levels of TNF-α expression among all control and case groups on the 28^th^ day of assay.

In the case of data acquired from quantitative assessment of TNF-α on the 56^th^ day of assay, it was demonstrated that the lowest and the highest levels of TNF-α expression are related to cases receiving both prebiotic bacteria (G4) groups, and control groups, respectively. Among cases receiving only one type of probiotic bacteria (G3, and G2) groups, and cases receiving both prebiotic bacteria (G4) groups, the highest levels of TNF-α expression was related to cases receiving only one type of probiotic bacteria (G2) groups and no significant difference was found. Identically, there was not found any significant difference for levels of TNF-α expression among zero, and cases receiving only one type of probiotic bacteria (G3, and G2) groups, and cases receiving both prebiotic bacteria (G4) groups on the 56^th^ day of assay. Of note, there was a significant difference for levels of TNF-α expression between control, and zero groups, and between control, and cases receiving only one type of probiotic bacteria (G3) groups, and cases receiving both prebiotic bacteria (G4) groups, as well.

With regard to data acquired from quantitative assessment of TNF-α on the 60^th^ day of assay, it was shown that the lowest and the highest levels of TNF-α expression with a significant difference are related to zero, and control groups, respectively. No significant difference was found among control groups, and cases receiving only one type of probiotic bacteria (G3, and G2) groups, and cases receiving both prebiotic bacteria (G4) groups on the 60^th^ day of assay. Among three receiving prebiotic bacteria (G2, G3, and G4), the highest levels of TNF-α expression are related to cases receiving only one type of probiotic bacteria (G3) group with no significant difference. Usage of probiotics did not show significant effects on levels of TNF-α expression in intestinal tissues of *Zebrafish* models infected by *Aeromonas hydrophila* on the 60^th^ day of assay.

### Biometric assessment of the *Zebrafish* models through weekly investigation of mortality rate, and height, and body weight in 14^th^, 28^th^, and 56^th^ day of assay

**Fig. 8:**
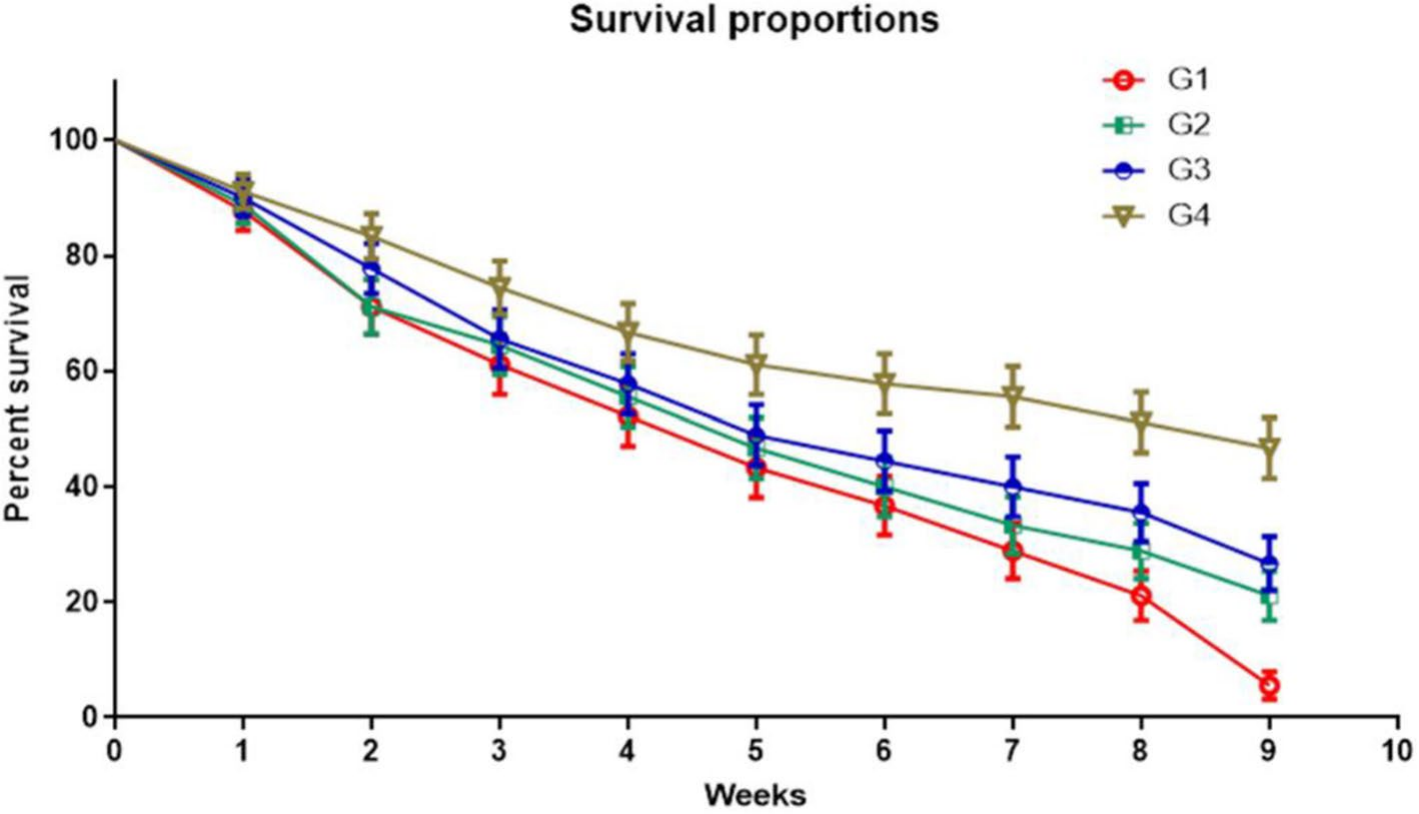
Investigation of survival proportions in the fish during nine weeks of the assay

**Fig. 9:**
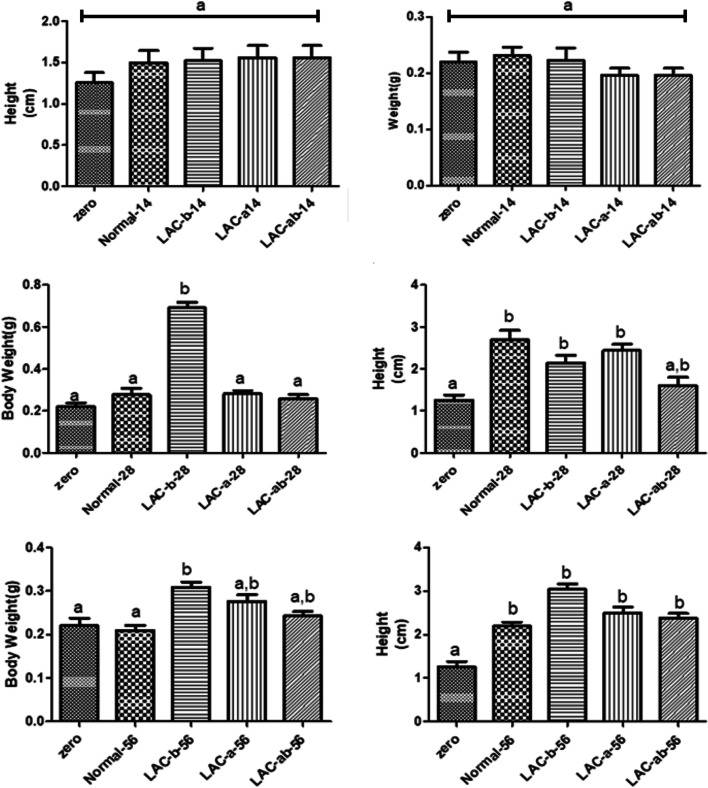
Investigation of height and bodyweight of the Fish in A) 28^th^, B) 56^th^, and C) 60^th^ day of assay

Figure 8, and 9, show results related to biometric assessment of *Zebrafish* models infected by *Aeromonas hydrophila* through investigation of parameters as mortality rate (weekly presented), height, and body weight in all case and control groups (G1, G2, G3, and G4) in 14^th^, 28^th^, and 56^th^ day of assay.

#### Biometric assessment of the *Zebrafish* through investigation of mortality rate

Figure 8, shows results related to biometric assessment of *Zebrafish* models infected by *Aeromonas hydrophila* through investigation of parameters as mortality rate (weekly presented), in all case and control groups (G1, G2, G3, and G4) in during nine weeks of the assay.

As it was clear, a nine-week investigation of the mortality rate of *Zebrafish* models at the end of every week was recorded and results are presented by survival rate. The lowest survival rate of Zebrafish was related to control groups that were infected by *Aeromonas hydrophila* and receiving no probiotic bacteria (G1). The lowest survival rate (the highest mortality rate) was observed in G1 groups eight weeks before being exposed to *Aeromonas hydrophila* and with the same trend in the ninth week after being infected by it. Precisely, the highest survival rate (the lowest mortality rate) of Zebrafish was observed in receiving both probiotic bacteria (G4) group in eight weeks before being exposed to *Aeromonas hydrophila* and with the same trend in the ninth week after being infected by it.

Totally, there was a decreasing trend for the survival rate of *Zebrafish* models in all control and case groups at the end of every week.

#### Biometric assessment of the Fish through Investigation of height and body weight in 14^th^ day of assay

Figure 9, shows results related to biometric assessment of *Zebrafish* models infected by *Aeromonas hydrophila* through investigation of parameters as height, and body weight in all case and control groups (G1, G2, G3, and G4) on the 14^th^, 28^th^, and 56^th^ day of assay.

According to Fig. 9, on the 14^th^ day of assay, there was not any significant difference for height and bodyweight of the fish among all control and case groups, proving that we need more time for investigation of positive effects on height and body weight after administration of probiotics.

According to Fig. 9, on the 28^th^ day of assay, the highest rate of body weight was recorded for groups receiving only one type of probiotic bacteria (G3) with a significant difference in comparison with zero, control, cases receiving only one type of probiotic bacteria (G2) groups, and cases receiving both prebiotic bacteria (G4) groups (*p* ≤ 0.05). But, there was not any significant difference among zero, normal, cases receiving only one type of probiotic bacteria (G2) groups, and cases receiving both prebiotic bacteria (G4) in case of body weight on the 28^th^ day of assay. In the case of height, although the highest rate of height was observed in the control group on the 28^th^ day of assay, the highest rate of height was also observed in cases receiving only one type of probiotic bacteria (G21) groups, among cases receiving bacteria (G2, G3, and G4) groups. There was not any significant difference among normal, cases receiving only one type of probiotic bacteria (G2) groups, cases receiving only one type of probiotic bacteria (G3) groups, and cases receiving both prebiotic bacteria (G2) in case of height on the 26^th^ day of assay. Interestingly, there was a significant difference for the height of the *Zebrafish* on the 28^th^ day of assay among zero, control, and both cases receiving only one type of probiotic bacteria (G3, and G2), proving profound effects of probiotics for the fish.

According to Fig. 9, on the 56^th^ day of assay, the highest rate of body weight was recorded for groups receiving only one type of probiotic bacteria (G3) with a significant difference in comparison with zero, and control groups (*p* ≤ 0.05). But, there was not any significant difference among zero, normal, cases receiving only one type of probiotic bacteria (G2) groups, and cases receiving both prebiotic bacteria (G4) in case of body weight on the 56^th^ day of assay. In the case of height, the highest rate of height was recorded for groups receiving only one type of probiotic bacteria (G3) with a significant difference in comparison with the zero group (*p* ≤ 0.05). In addition, there was not any significant difference among normal, cases receiving only one type of probiotic bacteria (G2) groups, and cases receiving both prebiotic bacteria (G4) in case of height on the 56^th^ day of assay.

## Discussion

It has been demonstrated that food-borne pathogens-associated diseases are of environmental, socio-economical, and clinical significance for aquaculture scientists, food microbiologists, medical microbiologists, environmentalists, and public health managers. The theory of “Probiotics for fostering gut health” has promisingly proved its clinical efficiency for humans [62–64]. Deciphering possible functional potentials of the microbiome of the biosphere was the reasonable method for selecting the desired mix of probiotics. One of the most important criteria for the selection of a probiotic especially an oral probiotic was choosing a Generally Recognized As Safe (GRAS) product which was defined by Food and Drug Administration (FDA).

It has been specified that LAB (majorly *lactobacillus*) has shown advantageous properties for induction of immunomodulatory and histologic effects on the fish as probiotic bacteria. Interestingly, they exist as intestinal microflora that makes researchers look up probiotics as supplementary food additives for the fish, and their crosstalk between probiotic-based diets and immune systems [25, 65–68].

As *Zebrafish* are the highly accepted animal for investigation of developmental biology, the most practical and well-established models for studying biological effects of probiotic bacteria, research related to aquatic nutrition, and intestinal inflammation, are selected to be our animal model.

This study aimed to investigate the antimicrobial effect, spatial distribution, histopathological effects of Lactobacillus acidophilus and Lactobacillus bulgaricus probiotics on Aeromonas hydrophila and to evaluate the expression of IL-1β and TNF-α cytokines in the Zebrafish model. Aeromonas hydrophila has been selected as a pathogen to investigate and prove the antimicrobial effects of probiotics against this pathogen. To prove the anti-inflammatory role of probiotics before and after the presence of the pathogen, the expression of IL-1β and TNF-α cytokines was assessed using real-time PCR at different time intervals. The role of probiotics in changes in height and weight, as well as mortality of fish in different periods, was also identified.s

Histopathological examinations at different times showed that in the group of fish receiving both probiotics on days 28 and 56, the growth and number of villus in the intestine, especially in the middle and distal regions, was higher than other groups. In this group, the number of goblet cells was more than other groups and the induction of infection did not have much effect on changing the height of the villus.

Regarding the changes in the number of lymphocyte cells, it was found that the simultaneous use of both types of probiotics in increasing the number of these types of cells on day 56 in the proximal region compared to other groups had a significant increase. Therefore, it seems that the simultaneous use of both antibiotics has played an important role in the histopathological changes mentioned above. On the other hand, LD probiotic compared to LA probiotic showed better improvement in the amount of desired indicators, including villus height and the number of goblet cells in some areas of the intestine, especially on day 56.

A significant increase in the height of intestinal villus and a decrease in the pathological effects of exposure to the pathogen in probiotic groups might be because the lactobacilli in the large intestine do not ferment indigestible carbohydrates and decrease the pH. This reduction leads to the survival of fast acid microorganisms, growth inhibition and binding of factors such as opportunistic pathogens. Similar studies by Pirarat et al. Have shown that the length of intestinal villus has increased in all sections, especially in the middle and upper sections, which was similar to the results of the present study [27].

Histological examination of the intestine during the period of probiotic consumption has shown that an increase in goblet cells occurs in the intestine of probiotic-fed fish, which leads to increased mucus production. In the present study, it was found that feeding with probiotics can increase the number of goblet cells in the intestine and reduce the symptoms of Aeromonas hydrophila.

One of the possible mechanisms in increasing the length of intestinal villus can be that when probiotics enter the stomach, it grows there and uses the available sugars to make a variety of short-chain fatty acids, which play an important role in increasing the length of intestinal villus.

In the case of acidophilus probiotics, treating with both probiotics showed the highest specific proliferation after 28, 56, and 60 days. The results obtained for LD probiotics show the highest proliferation, especially after induction of infection with the desired pathogen in the intestines of the fish. Other studies have shown that probiotics can work competitively to create the desired population and compete with endogenous microbes in the gut, using a variety of mechanisms including the production of inhibitory compounds, competition for chemicals or energy, and competition for adhesion sites [26]. Probiotics are able to fight against a variety of pathogenic bacteria by establishing colonies on the mucosal surface and increasing the population of beneficial bacteria in the host gastrointestinal tract and altering the bacterial flora. It has also been shown that the use of probiotics can alter the bacterial flora of the host intestine by increasing the growth of beneficial bacteria. One of the proposed mechanisms was that probiotics delay the binding or growth of pathogens in the intestinal mucosa of fish.

If both probiotics were used simultaneously on different days, the expression of both cytokines was lower than in other groups. The results show that the use of probiotics does not have a significant effect on increasing the level of inflammatory cytokines, even if exposed to a pathogen. But in general, the use of probiotics alters the expression of inflammatory cytokines. Considering the zebrafish as a model, these results can be generalized to humans.

The results showed that the use of probiotics in the diet has increased the life of fish and their weight, which was a significant increase compared to group zero. Weight enhancement was seen in all three groups receiving probiotics, which on day 56, the group that received LD had the highest weight, although this increase was not significant compared to the other two groups.

These results proved that the use of different probiotics in this experiment had a good effect on increasing the weight of fish. One of the reasons for these changes could be the role of probiotics in improving the natural intestinal flora and on the other hand the secretion of enzymes that increase the digestibility of food and break down indigestible compounds and thus increase appetite in aquatic. The same conclusion can be considered fr the high of the fish. In this case, receiving probiotics could have a positive effect on increasing the height of fish during the period of use.

One of the most important issues in this study was the effect of probiotics in reducing fish mortality, especially after exposure to the pathogen. Comparison of mortality results before and after exposure to the pathogen proves that in the group that did not receive probiotics, the survival rate decreased significantly, which was different from other groups, especially those that received probiotics. These results prove the favorable effect of probiotics in reducing fish mortality, especially after pathogen induction. Several mechanisms have been suggested that probiotics increase weight and thus improve growth function and ultimately improve the immune system in some aquatic animals. Probiotics by producing detoxifying compounds, breaking down indigestible particles, increasing appetite, increasing the absorption of minerals and trace elements, producing important digestive enzymes, and stimulating appetite.

Several studies are published regarding the effect of probiotics on fish health. In an experimental study done by *Qin et el.*, development and immunity response on *Zebrafish* treated with *Lactobacillus casei BL23* was investigated 14 and 35 days post-fertilization. They concluded that higher final body weight was observed at 14, and 35 days post-fertilization ((*P* < 0.05), and (*P* < 0.01), respectively) [69].

Another newly conducted experimental study by *Taida Juliana Adorian *et al. reported that case groups including fish fed with supplementary diets including 1 × 10^6^ CFU g^−1^ of both aforementioned probiotic bacteria showed significantly better growth, higher weight, higher length, higher weight gain, and higher protein levels than those fed the basal diet (control). Regardless of the concentration of administered probiotics, a higher survival rate was observed in the case group including fish fed with supplementary diets of both probiotics. Additionally, they reported that administration of 1 × 10^6^ CFU g^−1^ of both probiotic Bacillus drastically reduced hepatic inflammation through lowering levels of hepatic enzymes (AST, ALT, ALP) [1].

Another study by *Hai-peng Zhang *et al*.* was aimed at the evaluation of growth, immunity, and disease resistance in *koi carp* infected by *Aeromonas veronii TH0426* after treatment with different concentrations of *Lactobacillus Plantarum C20015,* for six weeks. They reported a specific growth rate, a higher percentage of weight gain for the fish, promoted food intake, the higher appetite of the fish, exacerbated enzymatic activation of superoxide dismutase (SOD), peroxidase (POD), lysozyme (LZM), and more levels of IgM antibody especially after 28 days of the assay in the experimental groups supplementarily fed with sprayed *Lactobacillus Plantarum C20015* than in the control. These improved activities, especially enzymatic activity, percentage of weight gain, and higher survival rate were reached in the concentration of 1 × 10^8^ CFU g^−1^ in comparison with cases fed with other concentrations of probiotic, and control groups [70]. As it has been reported, they represented the concentration of 1 × 10^8^ CFU g^−1^ of *Lactobacillus Plantarum C20015* as an acceptable probiotic candidate with enhanced immunoprotective efficacy and improved non-specific and specific immunity in *koi carp* to be used in fisheries industries.

Another experimental study conducted by *M. Soltani *et al*.,* indicated the highest levels in variables including hematological indices, weight gain, final weight, condition factor, feed utilization, thermal unit growth coefficient, and survival rate, and the lowest food conversion ratio in groups that were fed with 1 × 10^8^ CFU g^−1^ of *Lactobacillus Plantarum* and administered normal diet (vaccinated with *streptococcosis and lactococcosis*) in the same time.

In another study conducted by *Y. Wang. *et al*.,* it was reported that there are immunoprotective roles as reduced degranulation of mast cells, a remarkable decrease in expression of IL-1β and increase in IL-10 expression (post-challenge related data), restored intestinal morphology, sustainable barrier function, and a higher percentage of intraepithelial lymphocytes related to the usage of highly adhesive strains of *Bacillus coagulans 09.712*, and *Lactobacillus Plantarum 08.923* when they are orally administrated [71].

Similarly, in one study, *LA* induced macrophage-mediated inflammatory responses against *Aeromonas hydrophila*, a significant increase in TNF-α expression, and attenuated *Aeromonas hydrophila* induced apoptosis was reported in freshwater carp *Catla catla* after being fed with *LA* as probiotic bacteria [72].

Conclusively, there is a wide range of studies, indicating beneficial immunomodulatory effects on immune responses, weight gain, pro-inflammatory cytokines (down-regulatory effects on TNF-α, IL-1β, IL-6, and IL-12 expression, and up-regulatory effects on IL-10 and TGF-β expression), and increased survival rate by LAB (*Lactococcus lactis, Lactobacillus fermentum, LA, Bacillus subtilis* strains) on food-borne pathogens like *Aeromonas hydrophila* infecting common carp fish (*Cyprinus carpio*), suggesting usage of such potentiated probiotics in aquaculture [65, 73, 74]. Results of all mentioned studies, apart from the type of probiotic and the fish, are in good agreement with our results in case of improvement in body weight, immune responses against infection, length of intestinal villus, and survival rate through the usage of probiotic bacteria used for fish industries.

## Future directions and conclusion

According to the high mortality and morbidity rate, and the unsuccessfulness of antibiotics or other therapeutic strategies for the fish, food microbiologists, and health managers will look up more efficient approaches. One of the criteria for choosing them is not having any disadvantage for a health situation. We believe that usage of probiotics, as live microorganisms with proven immunomodulatory effects on intestinal tissues, can pave the path for a reduction in microbial infections in fisheries, increase in survival rate, decrease in the emergence of antibiotic resistance and their direct consequences, and diminution in the transmission of resistant pathogens from marine environments to human gut through the food chain.

## Data Availability

All data generated or analysed during this study are included in this published article.
